# Prediction of Renal Prognosis in Patients with Autosomal Dominant Polycystic Kidney Disease Using *PKD1/PKD2* Mutations

**DOI:** 10.3390/jcm9010146

**Published:** 2020-01-05

**Authors:** Hiroshi Kataoka, Hinata Fukuoka, Shiho Makabe, Rie Yoshida, Atsuko Teraoka, Yusuke Ushio, Taro Akihisa, Shun Manabe, Masayo Sato, Michihiro Mitobe, Ken Tsuchiya, Kosaku Nitta, Toshio Mochizuki

**Affiliations:** 1 Department of Nephrology, Tokyo Women’s Medical University, Tokyo 162-8666, Japan; kataoka@twmu.ac.jp (H.K.); makabe.shiho@twmu.ac.jp (S.M.); dalieyoshi@gmail.com (R.Y.); terako320@yahoo.co.jp (A.T.); ushio.u_chan@icloud.com (Y.U.); taro09071031@gmail.com (T.A.); shunmn5711@gmail.com (S.M.); sato.masayo@twmu.ac.jp (M.S.); fmitobe@d1.dion.ne.jp (M.M.); knitta@twmu.ac.jp (K.N.); 2 Clinical Research Division for Polycystic Kidney Disease, Department of Nephrology, Tokyo Women’s Medical University, Tokyo 162-8666, Japan; 3 Tokyo Women’s Medical University, Tokyo 162-8666, Japan; hinata.fukuoka@gmail.com; 4 Department of Blood Purification, Tokyo Women’s Medical University, Tokyo 162-8666, Japan; tsuchiya@twmu.ac.jp

**Keywords:** polycystic kidney disease, *PKD1*, *PKD2*, mutation, renal prognosis, renal progression

## Abstract

Autosomal dominant polycystic kidney disease (ADPKD) patients with *PKD1* mutations, particularly those with truncating mutations, show poor prognosis. However, the differences in disease progression with different mutation types are unclear. Here, a comparative study was conducted on the renal prognosis of patients with ADPKD who were categorized based on genotype (*PKD1* versus *PKD2* mutation), mutation type (truncating mutation: nonsense, frameshift, splicing mutation, and large deletion; non-truncating mutation: substitution and in-frame deletion), and mutation position. A total of 123 patients visiting our hospital were enrolled. Renal prognosis was poor for those with *PKD1* splicing, *PKD1* frameshift, and *PKD2* splicing mutations. Despite the truncating mutation, the renal prognosis was relatively favorable for patients with nonsense mutations. Three out of five patients with *PKD2* mutation required renal replacement therapy before 58 years of age. In conclusion, we showed that renal prognosis differs according to mutation types in both *PKD1* and *PKD2*, and that it was favorable for those with nonsense mutations among patients with *PKD1* truncating mutations. It was also confirmed that renal prognosis was not always favorable in patients with *PKD2* mutations. A detailed assessment of mutation types may be useful for predicting the renal prognosis of patients with ADPKD.

## 1. Introduction

Autosomal dominant polycystic kidney disease (ADPKD), one of the most common hereditary kidney diseases, is characterized by the gradual growth of cysts in the kidneys. Studies have shown that approximately 50% of patients with ADPKD progress to end-stage renal failure by the age of 60 years [1]. Mutations in *PKD1* and *PKD2* have been identified to be responsible for the disease [2,3]. Polycystin 1 (PC1), encoded by *PKD1*, assists in the mechanosensing of urine flow by the cilia of the renal tubules. Polycystin 2 (PC2), encoded by *PKD2*, functions as a calcium channel in cooperation with PC1. PC1 and PC2 play critical roles in the suppression of renal tubule dilation, and the dysfunction of either gene may lead to renal tubule expansion and cyst formation [4]. Reportedly, approximately 80% of patients with ADPKD harbor *PKD1* mutations, 15% harbor *PKD2* mutations, and the remaining 5–10% are genetically unresolved or rare mutations in other genes, such as hepatocyte nuclear factor 1β (HNF1B) and neutral α-glucosidase AB (GANAB) [5]. As ADPKD is an autosomal dominant inherited disease, patients with ADPKD harbor one normal allele of *PKD1* or *PKD2,* and cysts are not formed if this allele is functional. Reports show that the loss of function of both alleles because of somatic mutations in the normal allele, in addition to germline mutations, initiates cyst formation [6,7].

Several reports indicate that the progression of the disease varies with genotype [8,9,10,11,12,13]. Patients with mutations in *PKD1* progress to end-stage renal disease (ESRD) between 53 and 67 years, whereas ESRD is reached between 69 and 79 years for those with *PKD2* mutations. Studies suggest that the age at ESRD varies by approximately 10–20 years between those with *PKD1* and *PKD2* mutations. Another study reported that among patients with mutations in *PKD1*, those with truncating mutations progressed to ESRD at the age of 55 years, while those with non-truncating mutations did so at 67 years [9]. However, the relationship between differences in disease progression and mutation types is unclear. In this study, we investigated the differences in renal prognosis among 123 patients with ADPKD based on genotype, mutation type, and mutation position.

## 2. Experimental Section

### 2.1. Study Design

Kidney survival was retrospectively examined in 123 ADPKD patients with identified *PKD1* or *PKD2* mutations. ADPKD was diagnosed using previously described criteria [14], and genetic analyses were performed, as reported previously [15]. First, among 110 ADPKD patients in which mutations were detected at the Tokyo Women’s Medical University Hospital, Japan, between November 2010 and June 2016, patients who could not be followed up (*n* = 2) were excluded from participation. The remaining 108 patients and 15 family members from 12 out of 108 pedigrees who attended our hospital were ultimately enrolled in the present study (Figure S1). Written informed consent was obtained from each participant. All procedures performed in studies involving human participants were in accordance with the 1964 Helsinki declaration and its later amendments or comparable ethical standards. This study was approved by the ethical committee of Tokyo Women’s Medical University (No. 196B). The dataset analyzed for this study is provided as supplementary material to make future comparisons feasible and reliable (Supplementary Data S1).

### 2.2. Mutation Analysis

Patients’ genomic DNA was extracted from peripheral blood lymphocytes using the QIAamp DNA blood maxi kit (Qiagen Inc., Hilden, Germany). Mutational analyses of *PKD1* and *PKD2* were performed using the Sanger method, next-generation sequence (NGS) method, and multiplex ligation-dependent probe amplification (MLPA) method. In 102 patients, mutation analyses had already been performed using NGS, Sanger sequencing in exon 1 of *PKD1*, or MLPA, as reported previously [15]. In the remaining eight patients, the regions containing each exon and splice junctions of *PKD1* and *PKD2* were amplified using polymerase chain reaction (PCR) and were sequenced using the Sanger method, as described previously (Table S1) [16,17,18].

### 2.3. Classification of Mutation Types

Frameshift mutations, nonsense mutations, splicing mutations, and large rearrangements were classified as truncating mutations, whereas in-frame insertions or deletions (indels) and substitutions were classified as non-truncating mutations.

### 2.4. Classification of Mutation Positions

The mutation positions of *PKD1* were examined separately for the *PKD1* N-terminal domain (cDNA nucleotide position; 1-2552), PKD domain (2553-6435), REJ (receptor for egg jelly) domain (6438-9180), TM (transmembrane) domain (9225-12318), and *PKD1* C-terminal domain (12319-12906).

### 2.5. Outcome Evaluation (End-Point)

The outcome of interest was progression to renal replacement therapy (RRT), which was defined as the initiation of chronic dialysis or kidney transplantation. The subjects were followed up until June 2018.

### 2.6. Evaluation of Age at RRT

We evaluated the age at RRT between patients with *PKD1* and *PKD2* mutations, and among patients with various types of mutations in each gene.

### 2.7. Statistical Analysis

Continuous variables are reported as medians (minimum–maximum), and categorical variables are reported as percentages unless otherwise stated. We compared participant outcomes by performing a chi-square test or Fisher’s exact test. Prognostic variables for renal outcome were assessed using the univariate and multivariate Cox proportional hazards method. Variables with *p*-values < 0.1 in the univariate model were included in the multivariate analyses. Analyses were examined using two models. Model 1 examined all variables as the independent variables. Model 2 examined the outcomes using *PKD1* splicing as the reference category. Time from birth to RRT was computed using the Kaplan–Meier method and evaluated using the log-rank test. *p*-values < 0.05 were considered statistically significant. All statistical analyses were performed using the JMP Pro ver.14.1.0 software program (SAS Institute, Cary, NC, USA).

## 3. Results

### 3.1. Patients’ Characteristics

The characteristics of the entire cohort at baseline with *PKD1* and *PKD2* mutations are shown in Table 1. The 123 subjects consisted of 52 men (42.3%) and 71 women (57.7%). Ninety-nine patients harbored *PKD1* mutations (80.5%) and 24 harbored *PKD2* mutations (19.5%). Truncating mutations were present in 74.0% cases and non-truncating mutations were present in 26.0% of cases. The truncating mutations were detected in 69.7% of patients with *PKD1* mutations and 91.7% of patients with *PKD2* mutations (*p* = 0.0277). Furthermore, the proportion of nonsense mutations in patients with *PKD2* mutations was significantly higher than that in patients with *PKD1* mutations (*p* = 0.0158; *PKD1* versus *PKD2* = 28.3% versus 54.2%). Patient characteristics stratified by sex are shown in Table S2. There were no significant differences between men and women regarding the ratio of mutation type.

### 3.2. Mutation Types as Renal Prognostic Indicators in Patients with ADPKD

#### 3.2.1. Influence of the Mutated Gene on Renal Outcome (Age at RRT)

The median (minimum–maximum) follow-up period from birth for all patients was 50 (20–86) years. At the follow-up examination in June 2018, 37 (30.1%) out of 123 patients required RRT; RRT was required by 32 (32.3%) out of 99 patients with *PKD1* mutations and five (20.8%) out of 24 patients with *PKD2* mutations, and the median (minimum–maximum) age of patients who required RRT was 52 (34–79) years for patients with *PKD1* mutations and 51 (44–73) years for patients with *PKD2* mutations. Five out of 32 patients with mutations in *PKD1* required RRT after 68 years of age. Three out of five patients with mutations in *PKD2* required RRT before 58 years of age (Table S3).

In patients with *PKD1* mutations, RRT was required for 24 (34.8%) out of 69 patients with *PKD1* truncating mutations (*PKD1*T) and eight (26.7%) out of 30 patients with *PKD1* non-truncating mutations (*PKD1*NT) until June 2018, and the median (minimum–maximum) age of patients who required RRT was 52 (34–72) years in patients with *PKD1*T and 57 (44–79) years in patients with *PKD1*NT. A detailed assessment of mutation types showed that RRT was required for 11 (44.0%) out of 25 patients with *PKD1* frameshift mutation, five (45.5%) out of 11 patients with *PKD1* splicing mutation, one out of five patients with *PKD1* large deletion mutation, seven (25.0%) out of 28 patients with *PKD1* nonsense mutation, and eight (29.6%) out of 28 patients with *PKD1* substitution mutation, and the median (minimum–maximum) age of patients who required RRT was 48 (34–64) years in patients with *PKD1* frameshift mutations, 49 (42–65) years in patients with *PKD1* splicing mutations, 62 (45–72) years in patients with *PKD1* nonsense mutations, 47 years in a patient with a *PKD1* large deletion mutation, and 57 (44–79) years in patients with *PKD1* substitution mutations.

**Figure 1:**
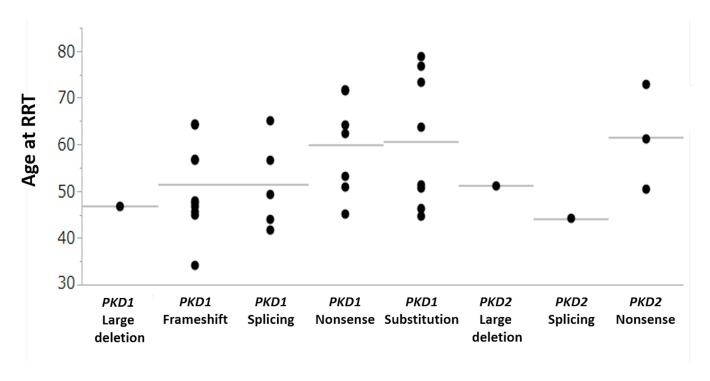
Age at RRT. RRT = renal replacement therapy.

In patients with *PKD2* mutations, truncating mutations were present in 22 patients out of 24 patients with PKD2 mutations and all mutations in five patients with *PKD2* who required RRT were truncating mutations. RRT was needed for the one patient with a *PKD2* splicing mutation, three patients with *PKD2* nonsense mutations, and one patient with a *PKD2* large deletion mutation, and the ages of patients who required RRT were 44 years in a patient with a *PKD2* splicing mutation, 50, 61, and 73 years in patients with *PKD2* nonsense mutations, and 51 years in a patient with a *PKD2* large deletion mutation (Figure 1).

#### 3.2.2. Influence of Mutation Type on Renal Prognosis in the Entire Cohort

The univariate Cox regression analyses in the entire cohort showed that *PKD1* (hazard ratio (HR) = 2.75, 95% CI (1.16–8.10), *p* = 0.0202) and *PKD1*T (HR = 2.77, 95% CI (1.36–5.98), *p* = 0.0046) mutations were significantly associated with RRT (Table 2). Regarding a detailed assessment of mutation types, the sex-adjusted multivariate Cox regression analyses in the entire cohort showed that *PKD1* splicing (HR = 5.39, 95% CI (1.70–14.51), *p* = 0.0063), *PKD1* frameshift (HR = 3.14, 95% CI (1.43–6.59), *p* = 0.0055), and *PKD2* splicing (HR = 52.40, 95% CI (2.49–446.52), *p* = 0.0181) mutations were significantly associated with RRT (Table 2). When *PKD1* splicing was used for the reference category, the sex-adjusted multivariate Cox regression analysis in the entire cohort showed that *PKD1* nonsense (HR = 0.26, 95% CI (0.08–0.86), *p* = 0.0304), *PKD1* substitution (HR = 0.20, 95% CI (0.06–0.66), *p* = 0.0081), and *PKD2* nonsense (HR = 0.12, 95% CI (0.03–0.53), *p* = 0.0055) mutations were significantly less associated with RRT than *PKD1* splicing mutation (Table 3).

**Figure 2:**
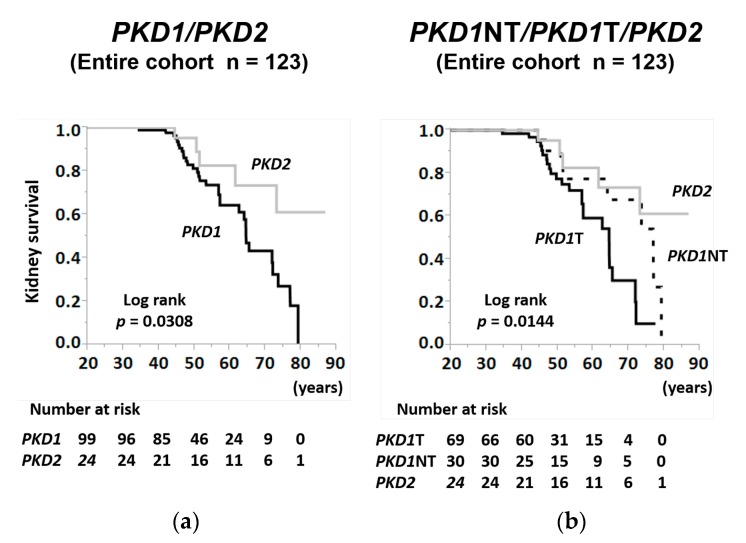
Kidney survival rate of patients with autosomal dominant polycystic kidney disease (ADPKD) stratified by genotypes and mutation types in the entire cohort. (**a**) The kidney survival rates of ADPKD patients with *PKD1* mutation and *PKD2* mutation in the entire cohort (log-rank, *p* = 0.0308). (**b**) The kidney survival rates of ADPKD patients with *PKD1*T, *PKD1*NT, and *PKD2* mutations in the entire cohort (log-rank, *p* = 0.0144). The number of patients at risk for progression to RRT at each time point is mentioned below the figures. *PKD1*T = *PKD1* truncating; *PKD1*NT = *PKD1* non-truncating.

The age-specific Kaplan–Meier method showed that the kidney survival rate of ADPKD patients with *PKD1* mutations was significantly lower than that of those with *PKD2* mutations in the entire cohort (Figure 2a; log-rank, *p* = 0.0308). When the *PKD1* cohort was divided into truncating and non-truncating mutations, the kidney survival rate was the lowest in patients with *PKD1*T (Figure 2b; log-rank, *p* = 0.0144).

**Figure 3:**
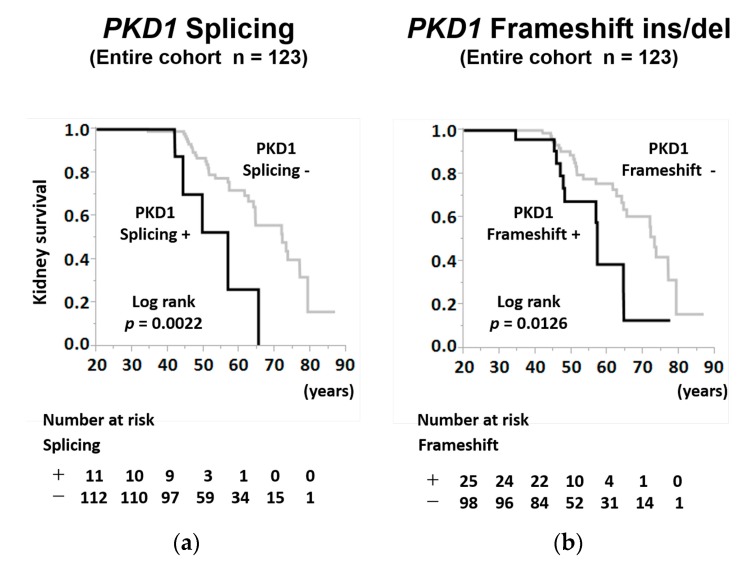
Kidney survival rate of patients with ADPKD stratified by *PKD1* splicing mutation and a *PKD1* frameshift mutation in the entire cohort. (**a**) The kidney survival rates of ADPKD patients with *PKD1* splicing mutation or without *PKD1* splicing mutation in the entire cohort (log-rank, *p* = 0.0022). (**b**) The kidney survival rates of ADPKD patients with *PKD1* frameshift mutation or without *PKD1* frameshift mutation in the entire cohort (log-rank, *p* = 0.0126). The number of patients at risk for progression to RRT at each time point is mentioned below the figures. ins/del = insertion/deletion.

The age-specific Kaplan–Meier survival curves indicated that the kidney survival rates of patients with *PKD1* splicing mutations were significantly lower than those of patients without *PKD1* splicing mutations in the entire cohort (Figure 3a; log-rank, *p* = 0.0022). Moreover, the age-specific Kaplan–Meier survival curves indicated that the kidney survival rates of patients with *PKD1* frameshift mutations were significantly lower than those of patients without *PKD1* frameshift mutations in the entire cohort (Figure 3b; log-rank, *p* = 0.0126).

#### 3.2.3. Influence of Mutation Type and Position on Renal Prognosis in PKD1 Truncating Cohort

In the subgroup analysis of the *PKD1*T cohort, the univariate Cox regression analyses showed that the *PKD1* nonsense mutation was a favorable prognostic factor for RRT (HR = 0.36, 95% CI (0.14–0.85), *p* = 0.0189), while the mutation position did not have any effect on renal prognosis (Table 4). When *PKD1* splicing was used for the reference category, the univariate Cox regression analysis in the *PKD1*T cohort showed that *PKD1* nonsense (HR = 0.24, 95% CI (0.07–0.81), *p* = 0.0236) mutation was significantly less associated with RRT than *PKD1* splicing mutation (Table 4).

**Figure 4:**
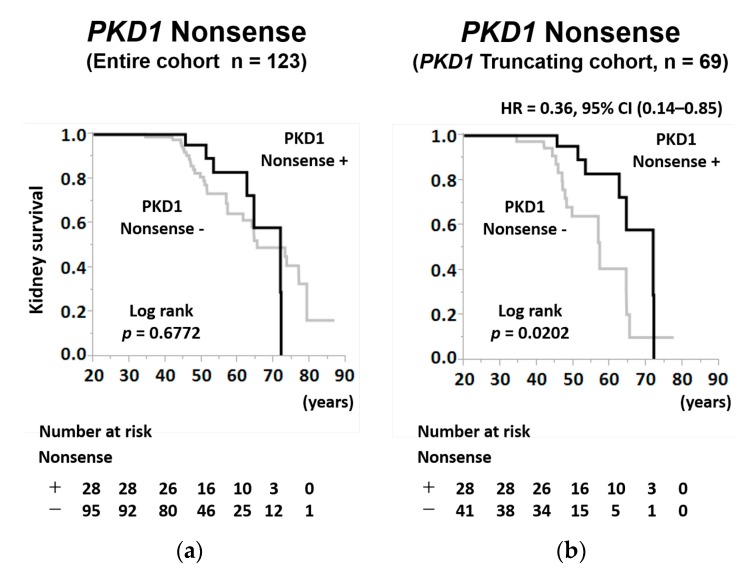
Kidney survival rate of patients with ADPKD stratified by *PKD1* nonsense mutation in the entire and *PKD1* truncating cohort. (**a**) The kidney survival rates of ADPKD patients with *PKD1* nonsense mutation or without *PKD1* nonsense mutation in the entire cohort (log-rank, *p* = 0.6722). (**b**) The kidney survival rates of ADPKD patients with *PKD1* nonsense mutation or without *PKD1* nonsense mutation in the *PKD1* truncating cohort (log-rank, *p* = 0.0202). The number of patients at risk for progression to RRT at each time point is mentioned below the figures; HR = hazard ratio; CI = confidence interval.

In the age-specific Kaplan–Meier analysis, although there was no significant difference among patients with *PKD1* nonsense mutations in the entire cohort (Figure 4a; log-rank; *p* = 0.6772), the kidney survival rate of patients with *PKD1* nonsense mutations was significantly higher than that of patients without *PKD1* nonsense mutations in the *PKD1T* cohort (Figure 4b; log-rank; *p* = 0.0202).

#### 3.2.4. Influence of Mutation Type on Renal Prognosis in the Cohort Stratified by Sex

To examine the sex-specific differences in the renal prognosis of patients with ADPKD, we conducted Cox regression analyses using stratification by sex. The univariate Cox regression analyses in the sex-stratified cohort showed that *PKD1* (HR = 3.64, 95% CI (1.02–23.23), *p* = 0.0462) and *PKD1* splicing mutation (HR = 24.45, 95% CI (3.19–149.12), *p* = 0.0049) in women, and *PKD1* truncating (HR = 4.02, 95% CI (1.38–14.75), *p* = 0.0093), *PKD1* frameshift (HR = 2.85, 95% CI (1.03–7.47), *p* = 0.0449), *PKD1* substitution (HR = 0.17, 95% CI (0.01–0.85), *p* = 0.0272), and *PKD2* splicing (HR = 22.42, 95% CI (1.04–234.30), *p* = 0.0478) mutations in men were significantly associated with RRT (Table 5).

## 4. Discussion

This study analyzed differences in renal prognosis in patients with ADPKD by genotype, mutation type, and mutation position. From among these factors, no significant difference in renal prognosis was observed by mutation position; however, a significant difference was observed by genotype and mutation type, particularly mutation types such as frameshift mutations, nonsense mutations, and splicing mutations.

Several studies have reported differences in renal prognosis by genotype [8,9,10,11,12,13,19]. The kidney survival rate depends on the causal genes (*PKD1*, *PKD2*) and differences between truncating or non-truncating mutations. Patients with *PKD1* mutations, particularly those with truncating mutations, show poor prognosis [8,9,10,11,12,13,19]. However, the relationship between differences in disease progression and mutation types, such as nonsense, frameshift, splicing mutation, and large deletions as truncating mutations, substitution, and in-frame deletion as non-truncating mutations, is unknown. In this study, we observed a significant difference between *PKD1* and *PKD2* mutations in terms of the proportion of patients who progressed to RRT within each genotype (32.3% in *PKD1*; 20.8% in *PKD2*), suggesting that renal prognosis is poorer in patients with mutations in *PKD1* than in those with *PKD2* mutations. This could be because the frequency of somatic mutations in *PKD1* is higher than that in *PKD2*, as *PKD1* is 4.4 times larger than *PKD2* [20]. Therefore, a larger number of cysts are formed in ADPKD patients with *PKD1* mutations, leading to poor renal prognosis [20,21].

In the present study, both Cox analyses and Kaplan–Meier curves showed that renal prognosis was poorer in patients with truncating mutations than in those with non-truncating mutations in *PKD1*. Several reports suggest that when mutation types are compared, progression in patients with truncating mutations is faster than that in those with non-truncating mutations. Rossetti et al. evaluated kidney survival by the age of reaching ESRD and observed no significant difference between the mutation types [13]. However, subsequent reports indicated that the renal prognosis of patients with truncating mutations is poor [9,10,19,22,23]. Furthermore, a study in which a knock-in mouse model of p.R3277C (RC) was used showed that progression was slower in *PKD1*^RC/RC^ mice than in *PKD1*^RC/null^ ones [24]. This occurs because non-truncating mutations, including substitution mutations, partially retain the function of the *PKD1* gene as a hypomorphic allele. In other words, cyst formation starts when the expression of polycystins is below the threshold. Polycystins are still functional to a limited extent in patients with non-truncating mutations, and their expression becomes lower than the threshold after a long period in such cases. This is possibly responsible for the relatively favorable prognosis of patients with non-truncating mutations.

When we evaluated renal prognosis by mutation types, it was poor in patients with *PKD1* splicing, *PKD1* frameshift, and *PKD2* splicing mutations; however, unexpectedly, it was relatively favorable in patients with *PKD1* nonsense and *PKD2* nonsense mutations (Table 3 and Table 4, Figure 4b). Considering that in our study, the proportion of nonsense mutations in patients with PKD2 mutations (54.2%) was significantly higher than that in patients with PKD1 mutations (28.3%), we need to pay more attention to the effect of nonsense mutations on the renal prognostic differences between patients with PKD1 mutation and with PKD2 mutations.

In the present study, we found sex-specific differences in mutation types regarding the renal outcome in patients with ADPKD. In men, *PKD1* frameshift and *PKD2* splicing showed a significantly positive association with RRT, and in women, *PKD1* splicing mutation showed a strong positive association with RRT (Table 5). Although the reason for this difference is not clear, it might influence the sex-specific differences in renal prognosis for patients with ADPKD. Our study is the first report that compares renal prognosis by mutation types among *PKD1* truncating mutations and highlights that renal prognosis is poorer in patients with splicing and frameshift mutations than in patients with nonsense mutations.

The reason for the difference in prognosis among truncating mutations is not proved. One possibility is the involvement of nonsense mRNA-mediated decay (NMD), which leads to the degradation of transcripts containing premature termination codons (PTCs) [25,26]. It is generally considered that NMD occurs for truncating mutations that generate PTCs, including frameshift and nonsense mutations and some splicing mutations. The efficacy of NMD varies between individuals, leading to modifications of the disease outcome. Reports show that in ADPKD, NMD does not occur rapidly for *PKD1* mutants and that proteins are produced by the mutant gene [27]. Although the efficacy of NMD in each mutation type is unknown, the transcripts that escape from NMD may exert dominant-negative effects to decrease the dosage of normal polycystin, leading to cyst formation in ADPKD.

In this study, a comparison of only patients who progressed to RRT revealed no difference in the age of reaching RRT between patients with mutations in *PKD1* and those with mutations in *PKD2* (median 52 years versus 51 years). Barua et al. reported that no patient with *PKD1* mutations progressed to ESRD at the age of 68 years or older, and no patient with *PKD2* mutations progressed to ESRD at the age of 58 years or younger [11]. Meanwhile, another study has reported that 9.8% of patients with *PKD2* mutations progressed to ESRD by 60 years [12]. In this study, from among 32 patients with *PKD1* mutations who required RRT, five progressed to RRT at the age of 68 years or older, and from among five patients with *PKD2* mutations who required RRT, three progressed to RRT at the age of 58 years or younger. These observations suggested that the renal prognosis of patients with *PKD2* mutations was not always favorable compared to that of patients with *PKD1* mutations. Furthermore, renal prognosis in patients with nonsense mutations in *PKD1* or *PKD2* was relatively favorable in terms of the age of reaching RRT (Figure 1, Table S3).

While these findings may have several implications for patients with ADPKD, our study has several limitations. First, the study was observational in nature, and any observed associations do not prove causality. Second, only the patients’ mutated genes and mutation types were considered, and their characteristics during the follow-up period were not considered. Third, the sample size was relatively small; hence, further studies are required to confirm our findings in a large patient cohort.

## 5. Conclusions

In conclusion, we showed that a detailed assessment of mutation types might be useful for determining the renal prognosis of patients with ADPKD. The renal prognosis was poor in patients with *PKD1* splicing and frameshift mutations, but favorable in those with nonsense mutations among patients with truncating mutations. The prognosis was not always favorable in patients with *PKD2* mutations. Further investigations using large cohorts and the elucidation of mechanisms using animal models are required.

## Figures and Tables

**Table 1:** Patient characteristics.

Variables	Entire	*PKD1*	*PKD2*	*p*-Value
	*n* = 123	*n* = 99	*n* = 24	
Sex (Men)	52 (42.3)	42 (42.4)	10 (41.7)	0.9463
Mutation Type				
Truncating	91 (74.0)	69 (69.7)	22 (91.7)	0.0363
Splicing	12 (9.8)	11 (11.1)	1 (4.2)	0.4575
Frameshift ins/del	30 (24.4)	25 (25.3)	5 (20.8)	0.6511
Large deletion	8 (6.5)	5 (5.1)	3 (12.5)	0.1865
Nonsense	41 (33.3)	28 (28.3)	13 (54.2)	0.0158
Non-truncating	32 (26.0)	30 (30.3)	2 (8.3)	0.0363
Substitution	29 (23.6)	27 (27.3)	2 (8.3)	0.0499
In-frame ins/del	3 (2.4)	3 (3.0)	0 (0.0)	1.0000
Mutation Position				
*PKD1* N-terminal domain	NA	19 (19.2)	NA	NA
PKD domain	NA	18 (18.2)	NA	NA
REJ domain	NA	21 (21.2)	NA	NA
TM domain	NA	36 (36.4)	NA	NA
*PKD1* C-terminal domain	NA	5 (5.1)	NA	NA

Count data are expressed as *n* (%). Abbreviations: *n*, number; %, percentages; PKD, polycystic kidney disease; ins/del, insertion/deletion; REJ, receptor for egg jelly; TM, transmembrane; NA, not applicable.

**Table 2:** Univariate and multivariate analysis of risk factors associated with RRT (Entire cohort, *n* = 123; Model 1).

Variables	Univariate Analysis		Multivariate Analysis	
	Hazard Ratio (95% CI)	*p*-Value	Hazard Ratio (95% CI)	*p*-Value
*PKD1* (vs. *PKD2*)	2.75 (1.16–8.10)	0.0202	NA	NA
Mutation Type				
*PKD1* Truncating	2.77 (1.36–5.98)	0.0046	NA	NA
*PKD1* Non-truncating	0.72 (0.31–1.52)	0.4068	NA	NA
*PKD2* Truncating	0.44 (0.15–1.05)	0.0645	NA	NA
*PKD2* Non-truncating	5.191 × 10^−9^ (0–.)	0.0961	NA	NA
*PKD1* Splicing	4.01 (1.35–9.67)	0.0156	5.39 (1.70–14.51)	0.0063
*PKD1* Frameshift ins/del	2.43 (1.14–4.88)	0.0232	3.14 (1.43–6.59)	0.0055
*PKD1* Large deletion	1.14 (0.06–5.43)	0.8975	-	-
*PKD1* Nonsense	0.84 (0.33–1.83)	0.6725	-	-
*PKD1* Substitution	0.74 (0.31–1.57)	0.4515	-	-
*PKD1* In-frame ins/del	3.998 × 10^−8^ (0–.)	0.5121	-	-
*PKD2* Splicing	32.69 (1.62–255.79)	0.0293	52.40 (2.49–446.52)	0.0181
*PKD2* Frameshift ins/del	1.776 × 10^−9^ (0–.)	0.0258	NA	NA
*PKD2* Large deletion	1.29 (0.07–6.15)	0.8089	-	-
*PKD2* Nonsense	0.41 (0.10–1.17)	0.1035	-	-
*PKD2* Substitution	5.191 × 10^−9^ (0–.)	0.0961	-	-
*PKD2* In-frame ins/del	NA	NA	NA	NA
Men (vs. women)	1.50 (0.78–2.89)	0.2205	1.18 (0.59–2.36)	0.6395

Variables with *p*-values of less than 0.1 with a 95% CI in the univariate model and sex were included in the multivariate model. Abbreviations: *n*, number; CI, confidence interval; PKD, polycystic kidney disease; ins/del, insertion/deletion; REJ, receptor for egg jelly; TM, transmembrane; NA, not applicable.

**Table 3:** Univariate and multivariate analysis of risk factors associated with RRT (entire cohort, *n* = 123; Model 2).

Variables	Univariate Analysis		Multivariate Analysis	
	Hazard Ratio (95% CI)	*p*-Value	Hazard Ratio (95% CI)	*p*-Value
Mutation Type				
*PKD1* Truncating	1 (reference)		NA	NA
*PKD1* Non-truncating	0.46 (0.19–1.03)	0.0601	NA	NA
*PKD2* Truncating	0.31 (0.10–0.77)	0.0106	NA	NA
*PKD2* Non-truncating	2.953 × 10^−9^ (0–.)	0.0384	NA	NA
*PKD1* Splicing	1 (reference)		1 (reference)	
*PKD1* Frameshift ins/del	0.53 (0.19–1.71)	0.2709	0.58 (0.19–1.75)	0.3297
*PKD1* Large deletion	0.33 (0.02–2.11)	0.2684	0.38 (0.04–3.57)	0.4005
*PKD1* Nonsense	0.24 (0.07–0.81)	0.0234	0.26 (0.08–0.88)	0.0304
*PKD1* Substitution	0.19 (0.06–0.65)	0.0099	0.20 (0.06–0.66)	0.0081
*PKD1* In-frame ins/del	3.130 × 10^−10^ (0–.)	0.2747	3.720 × 10^−10^ (0–.)	0.9998
*PKD2* Splicing	10.20 (0.47–94.14)	0.1147	9.92 (0.90–109.16)	0.0607
*PKD2* Frameshift ins/del	2.820 × 10^−10^ (0–.)	0.0010	3.190 × 10^−10^ (0–.)	0.9990
*PKD2* Large deletion	0.38 (0.02–2.42)	0.3374	0.41 (0.05–3.61)	0.4213
*PKD2* Nonsense	0.11 (0.02–0.46)	0.0032	0.12 (0.03–0.53)	0.0055
*PKD2* Substitution	2.910 × 10^−10^ (0–.)	0.0060	2.930 × 10^−10^ (0–.)	0.9993
*PKD2* In-frame ins/del	NA	NA	NA	NA
Men (vs. women)	1.50 (0.78–2.89)	0.2205	1.21 (0.60–2.45)	0.5985

Variables with *p*-values of less than 0.1 with a 95% CI in the univariate model and sex were included in the multivariate model. Abbreviations: *n*, number; CI, confidence interval; PKD, polycystic kidney disease; ins/del, insertion/deletion; REJ, receptor for egg jelly; TM, transmembrane; NA, not applicable.

**Table 4:** Univariate analysis of risk factors associated with RRT (*PKD1* Truncating cohort, n = 69).

Variables	Univariate Analysis (Model 1)		Univariate Analysis (Model 2)	
	Hazard Ratio (95% CI)	*p*-Value	Hazard Ratio (95% CI)	*p*-Value
Mutation Type				
*PKD1* Splicing	2.69 (0.88–6.89)	0.0796	1 (reference)	
*PKD1* Frameshift ins/del	1.72 (0.75–3.88)	0.1953	0.60 (0.21–1.93)	0.3637
*PKD1* Large deletion	0.81 (0.04–3.99)	0.8350	0.35 (0.02–2.30)	0.3042
*PKD1* Nonsense	0.36 (0.14–0.85)	0.0189	0.24 (0.07–0.81)	0.0236
Mutation Position				
*PKD1* N-terminal domain	0.86 (0.24–2.36)	0.7798	1 (reference)	
PKD domain	1.08 (0.40–2.59)	0.8749	1.24 (0.35–4.90)	0.7440
REJ domain	1.01 (0.33–2.54)	0.9904	1.16 (0.30–4.83)	0.8241
TM domain	1.36 (0.55–3.17)	0.4903	1.43 (0.44–5.44)	0.5624
*PKD1* C-terminal domain	4.873 × 10^−9^ (0–.)	0.0927	5.877 × 10^−9^ (0–.)	0.1461

Abbreviations: RRT, renal replacement therapy; *n,* number; PKD, polycystic kidney disease; CI, confidence interval; ins/del, insertion/deletion; REJ, receptor for egg jelly; TM, transmembrane.

**Table 5:** Univariate analysis of risk factors associated with RRT in the cohort stratified by sex.

Variables	Univariate Analysis (Model 1) Men, *n* = 52		Univariate Analysis (Model 1) Women, *n* = 71	
	Hazard Ratio (95% CI)	*p*-Value	Hazard Ratio (95% CI)	*p*-Value
*PKD1* (vs. *PKD2*)	1.79 (0.58–7.76)	0.3331	3.64 (1.02–23.23)	0.0462
Mutation Type				
*PKD1* Truncating	4.02 (1.38–14.75)	0.0093	1.83 (0.70–5.04)	0.2196
*PKD1* Non-truncating	0.17 (0.01–0.85)	0.0272	1.31 (0.45–3.42)	0.5953
*PKD2* Truncating	0.75 (0.17–2.27)	0.6343	0.29 (0.05–1.05)	0.0598
*PKD2* Non-truncating	4.924 × 10^−9^ (0–.)	0.1691	1.453 × 10^−8^ (0–.)	0.4762
*PKD1* Splicing	2.02 (0.47–6.17)	0.3057	24.45 (3.19–149.12)	0.0049
*PKD1* Frameshift ins/del	2.85 (1.03–7.47)	0.0449	1.57 (0.44–4.45)	0.4494
*PKD1* Large deletion	6.69 (0.35–39.26)	0.1597	1.384 × 10^−8^ (0–.)	0.3100
*PKD1* Nonsense	0.79 (0.18–2.46)	0.7052	0.97 (0.27–2.87)	0.9596
*PKD1* Substitution	0.17 (0.01–0.85)	0.0272	1.37 (0.47–3.58)	0.5386
*PKD1* In-frame ins/del	NA	NA	3.883 × 10^−8^ (0–.)	0.5782
*PKD2* Splicing	22.42 (1.04–234.30)	0.0478	NA	NA
*PKD2* Frameshift ins/del	1.415 × 10^−8^ (0–.)	0.2985	4.158 × 10^−9^ (0–.)	0.0402
*PKD2* Large deletion	1.76 (0.10–9.08)	0.6164	1.453 × 10^−8^ (0–.)	0.4762
*PKD2* Nonsense	0.34 (0.02–1.68)	0.2202	0.60 (0.09–2.14)	0.4697
*PKD2* Substitution	4.924 × 10^−9^ (0–.)	0.1691	1.453 × 10^−8^ (0–.)	0.4762
*PKD2* In-frame ins/del	NA	NA	NA	NA

Abbreviations: *n*, number; CI, confidence interval; PKD, polycystic kidney disease; ins/del, insertion/deletion; REJ, receptor for egg jelly; TM, transmembrane; NA, not applicable.

## Supplementary Materials

The following are available online at https://www.mdpi.com/2077-0383/9/1/146/s1, Figure S1: Patient selection flowchart, Table S1: Mutations in *PKD1* and *PKD2* detected in eight ADPKD patients, Table S2: Patient characteristics stratified by sex, Table S3: Patients reaching RRT with mutations in *PKD1* and *PKD2,* Data S1: The datasets analyzed for the present study.

## References

[1] Grantham J.J. (2008). Clinical practice. Autosomal dominant polycystic kidney disease. N. Engl. J. Med..

[2] The European Polycystic Kidney Disease Consortium (1994). The polycystic kidney disease 1 gene encodes a 14 kb transcript and lies within a duplicated region on chromosome 16. Cell.

[3] Mochizuki T., Wu G., Hayashi T., Xenophontos S.L., Veldhuisen B., Saris J.J., Reynolds D.M., Cai Y., Gabow P.A., Pierides A. (1996). PKD2, a gene for polycystic kidney disease that encodes an integral membrane protein. Science.

[4] Nauli S.M., Alenghat F.J., Luo Y., Williams E., Vassilev P., Li X., Elia A.E., Lu W., Brown E.M., Quinn S.J. (2003). Polycystins 1 and 2 mediate mechanosensation in the primary cilium of kidney cells. Nat. Genet..

[5] Bergmann C., Guay-Woodford L.M., Harris P.C., Horie S., Peters D.J.M., Torres V.E. (2018). Polycystic kidney disease. Nat. Rev. Dis. Primers.

[6] Qian F., Watnick T.J., Onuchic L.F., Germino G.G. (1996). The molecular basis of focal cyst formation in human autosomal dominant polycystic kidney disease type I. Cell.

[7] Brasier J.L., Henske E.P. (1997). Loss of the polycystic kidney disease (PKD1) region of chromosome 16p13 in renal cyst cells supports a loss-of-function model for cyst pathogenesis. J. Clin. Investig..

[8] Hateboer N., Dijk M.A.V., Bogdanova N., Coto E., Saggar-Malik A.K., San Millan J.L., Torra R., Breuning M., Ravine D. (1999). Comparison of phenotypes of polycystic kidney disease types 1 and 2. European PKD1-PKD2 Study Group. Lancet.

[9] Cornec-Le Gall E., Audrezet M.P., Chen J.M., Hourmant M., Morin M.P., Perrichot R., Charasse C., Whebe B., Renaudineau E., Jousset P. (2013). Type of PKD1 mutation influences renal outcome in ADPKD. J. Am. Soc. Nephrol..

[10] Higashihara E., Horie S., Kinoshita M., Harris P.C., Okegawa T., Tanbo M., Hara H., Yamaguchi T., Shigemori K., Kawano H. (2018). A potentially crucial role of the PKD1 C-terminal tail in renal prognosis. Clin. Exp. Nephrol..

[11] Barua M., Cil O., Paterson A.D., Wang K., He N., Dicks E., Parfrey P., Pei Y. (2009). Family history of renal disease severity predicts the mutated gene in ADPKD. J. Am. Soc. Nephrol..

[12] Cornec-Le Gall E., Audrezet M.P., Renaudineau E., Hourmant M., Charasse C., Michez E., Frouget T., Vigneau C., Dantal J., Siohan P. (2017). PKD2-Related Autosomal Dominant Polycystic Kidney Disease: Prevalence, Clinical Presentation, Mutation Spectrum, and Prognosis. Am. J. Kidney Dis..

[13] Rossetti S., Burton S., Strmecki L., Pond G.R., San Millan J.L., Zerres K., Barratt T.M., Ozen S., Torres V.E., Bergstralh E.J. (2002). The position of the polycystic kidney disease 1 (PKD1) gene mutation correlates with the severity of renal disease. J. Am. Soc. Nephrol..

[14] Pei Y., Obaji J., Dupuis A., Paterson A.D., Magistroni R., Dicks E., Parfrey P., Cramer B., Coto E., Torra R. (2009). Unified criteria for ultrasonographic diagnosis of ADPKD. J. Am. Soc. Nephrol..

[15] Mochizuki T., Teraoka A., Akagawa H., Makabe S., Akihisa T., Sato M., Kataoka H., Mitobe M., Furukawa T., Tsuchiya K. (2019). Mutation analyses by next-generation sequencing and multiplex ligation-dependent probe amplification in Japanese autosomal dominant polycystic kidney disease patients. Clin. Exp. Nephrol..

[16] Tsuchiya K., Komeda M., Takahashi M., Yamashita N., Cigira M., Suzuki T., Suzuki K., Nihei H., Mochizuki T. (2001). Mutational analysis within the 3’ region of the PKD1 gene in Japanese families. Mutat. Res..

[17] Hayashi T., Mochizuki T., Reynolds D.M., Wu G., Cai Y., Somlo S. (1997). Characterization of the exon structure of the polycystic kidney disease 2 gene (PKD2). Genomics.

[18] Watnick T.J., Piontek K.B., Cordal T.M., Weber H., Gandolph M.A., Qian F., Lens X.M., Neumann H.P., Germino G.G. (1997). An unusual pattern of mutation in the duplicated portion of PKD1 is revealed by use of a novel strategy for mutation detection. Hum. Mol. Genet..

[19] Heyer C.M., Sundsbak J.L., Abebe K.Z., Chapman A.B., Torres V.E., Grantham J.J., Bae K.T., Schrier R.W., Perrone R.D., Braun W.E. (2016). Predicted Mutation Strength of Nontruncating PKD1 Mutations Aids Genotype-Phenotype Correlations in Autosomal Dominant Polycystic Kidney Disease. J. Am. Soc. Nephrol..

[20] Harris P.C., Bae K.T., Rossetti S., Torres V.E., Grantham J.J., Chapman A.B., Guay-Woodford L.M., King B.F., Wetzel L.H., Baumgarten D.A. (2006). Cyst number but not the rate of cystic growth is associated with the mutated gene in autosomal dominant polycystic kidney disease. J. Am. Soc. Nephrol..

[21] Rossetti S., Harris P.C. (2007). Genotype-phenotype correlations in autosomal dominant and autosomal recessive polycystic kidney disease. J. Am. Soc. Nephrol..

[22] Hwang Y.H., Conklin J., Chan W., Roslin N.M., Liu J., He N., Wang K., Sundsbak J.L., Heyer C.M., Haider M. (2016). Refining Genotype-Phenotype Correlation in Autosomal Dominant Polycystic Kidney Disease. J. Am. Soc. Nephrol..

[23] Liu B., Chen S.C., Yang Y.M., Yan K., Qian Y.Q., Zhang J.Y., Hu Y.T., Dong M.Y., Jin F., Huang H.F. (2015). Identification of novel PKD1 and PKD2 mutations in a Chinese population with autosomal dominant polycystic kidney disease. Sci. Rep..

[24] Hopp K., Ward C.J., Hommerding C.J., Nasr S.H., Tuan H.F., Gainullin V.G., Rossetti S., Torres V.E., Harris P.C. (2012). Functional polycystin-1 dosage governs autosomal dominant polycystic kidney disease severity. J. Clin. Investig..

[25] Kurosaki T., Maquat L.E. (2016). Nonsense-mediated mRNA decay in humans at a glance. J. Cell Sci..

[26] Nguyen L.S., Wilkinson M.F., Gecz J. (2014). Nonsense-mediated mRNA decay: Inter-individual variability and human disease. Neurosci. Biobehav. Rev..

[27] Rossetti S., Strmecki L., Gamble V., Burton S., Sneddon V., Peral B., Roy S., Bakkaloglu A., Komel R., Winearls C.G. (2001). Mutation analysis of the entire PKD1 gene: Genetic and diagnostic implications. Am. J. Hum. Genet..

