# Supplementary material for: Prediction of Renal Prognosis in Patients with Autosomal Dominant Polycystic Kidney Disease Using PKD1/PKD2 Mutations
Source: J Clin Med. 2020 Jan 5;9(1):146. doi: 10.3390/jcm9010146 (PMC7019244; doi:10.3390/jcm9010146)
Supplement: Supplementary file 1 [file jcm-09-00146-s001.pdf]

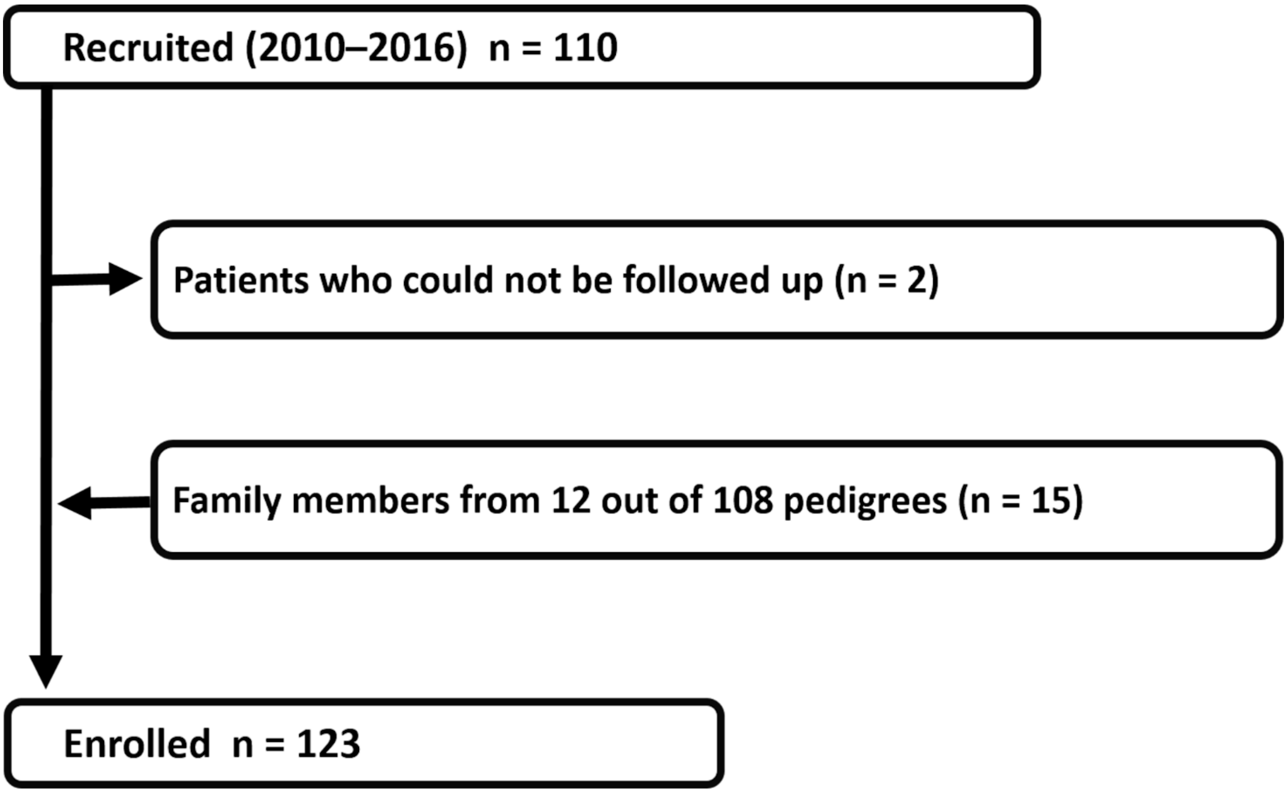

Figure S1. Patient selection flowchart. From 110 initially patients screened, 2 patients who could not be followed up were excluded from participation. The remaining 108 patients and 15 family members from 12 out of 108 pedigrees who attended our hospital were ultimately enrolled in the present study.

Supplementary Table S1. Patients with mutations in *PKD1* and *PKD2*

Patient number	Gene	Exon/IVS	cDNA change	Amino acid change	Mutation Type	PKD DB
120	*PKD1*	Exon 25	c.9051C>G	p.Tyr3017X	Nonsense	
16	*PKD1*	IVS 35	c.10618+1G>A	p.Gly3540fs	Splicing	
40	*PKD1*	Exon 40	c.11379delG	p.Thr3794fs31X	Frameshift	DP
46	*PKD1*	Exon 40	c.11379delG	p.Thr3794fs31X	Frameshift	DP
30	*PKD1*	Exon 40	c.12220_12221del CT	p.Leu4074fs82X	Frameshift	
13	*PKD2*	Exon 4	c.973C>T	p.Arg325X	Nonsense	DP
37	*PKD2*	Exon 5	c.1249C>T	p.Arg417X	Nonsense	DP
22	*PKD2*	Exon 13	c.2507_2508insA	p.Tyr836X	Frameshift	

Abbreviations: IVS, intervening sequence; PKDDB, polycystic kidney disease mutation database; ins, insertion; del, deletion; fs, frameshift; DP, definitely pathogenic

Supplementary Table S2. Patient characteristics stratified by sex

Variables	Entire	Men	Women	*p*-value
	n = 123	n = 52	n = 71	
PKD1	99 (80.5)	42 (80.8)	57 (80.3)	0.9463
PKD2	24 (19.5)	10 (19.2)	14 (19.7)	0.9463
Mutation Type (PKD1)				
Truncating	69 (56.1)	32 (61.5)	37 (52.1)	0.2981
Splicing	11 (8.9)	4 (7.7)	7 (9.9)	0.7585
Frameshift ins/del	25 (20.3)	13 (25.0)	12 (16.9)	0.2702
Large deletion	5 (4.1)	1 (1.9)	4 (5.6)	0.3951
Nonsense	28 (22.8)	14 (26.9)	14 (19.7)	0.3465
Non-truncating	30 (24.4)	10 (19.2)	20 (28.2)	0.2541
Substitution	27 (22.0)	10 (19.2)	17 (23.9)	0.5328
Inframe ins/del	3 (2.4)	0 (0.0)	3 (4.2)	0.2619
Mutation Type (PKD2)				
Truncating	22 (17.9)	9 (17.3)	13 (18.3)	1.0000
Splicing	1 (0.8)	1 (1.9)	0 (0.0)	0.4228
Frameshift ins/del	5 (4.1)	2 (3.9)	3 (4.2)	1.0000
Large deletion	3 (2.4)	2 (3.9)	1 (1.4)	0.5730
Nonsense	13 (10.6)	4 (7.7)	9 (12.7)	0.5544
Non-truncating	2 (1.6)	1 (1.9)	1 (1.4)	1.0000
Substitution	2 (1.6)	1 (1.9)	1 (1.4)	1.0000
Inframe ins/del	0 (0.0)	0 (0.0)	0 (0.0)	NA

Count data are expressed as n (%). Abbreviations: n, number; %, percentages; PKD, polycystic kidney disease; ins/del, insertion/deletion; REJ, Receptor for Egg Jelly; TM, transmembrane; NA, not applicable

Supplementary Table S3. Patients reaching RRT with mutations in *PKD1* and in *PKD2* ; n = 37

Pedigree number	Sex	Age at RRT	Gene	Mutation Type	Exon /IVS	cDNA change	Amino acid change
124	M	47	*PKD1*	Large deletion	IVS10 - Exon46	c.2098_30576del28478	p.Val700_Thr4302del3602
30	M	34	*PKD1*	Frameshift	40	c.12220_12221del CT	p.Leu4074fs82X
41	M	45	*PKD1*	Frameshift	15	c.5968_5969delAG	p.Arg1990fs58X
24	F	45	*PKD1*	Frameshift	15	c.5014_5015delAG	p.Arg1672fs97X
36	F	46	*PKD1*	Frameshift	15	c.6255delC	p.Pro2085fs30X
83	M	47	*PKD1*	Frameshift	15	c.5994_5995insCG	p.Gly1999fs117X
45	M	48	*PKD1*	Frameshift	11	c.2630_2631insCCCTG	p.Leu877fs22X
20	F	56	*PKD1*	Frameshift	25	c.9036_9037delGT	p.Thr3012fs55X
38	M	57	*PKD1*	Frameshift	40	c.11407_11408 delCTinsG	p.Leu3803fs22X
17	M	57	*PKD1*	Frameshift	19	c.7579_7580delGT	p.Val2527fs66X
7	F	64	*PKD1*	Frameshift	18	c.7422_7423insG	p.Gly2474fs26X
10	M	64	*PKD1*	Frameshift	15	c.3591_3592insTG	p.Ser1198X
107	M	42	*PKD1*	Splicing	IVS 39	c.11269+1G>C	p.Ala3757fs
107	F	44	*PKD1*	Splicing	IVS 39	c.11269+1G>C	p.Ala3757fs
102	F	49	*PKD1*	Splicing	IVS 4	c.529+1G>A	p.Gly177fs
16	M	56	*PKD1*	Splicing	IVS 35	c.10618+1G>A	p.Gly3540fs
39	M	65	*PKD1*	Splicing	IVS 37	c.11017-1G>C	p.Arg3672fs
26	M	45	*PKD1*	Nonsense	11	c.2788G>T	p.Glu930X
76	M	51	*PKD1*	Nonsense	44	c.12010C>T	p.Gln4004X
120	F	53	*PKD1*	Nonsense	25	c.9051C>G	p.Tyr3017X
89	F	62	*PKD1*	Nonsense	5	c.679C>T	p.Gln227X
32	M	64	*PKD1*	Nonsense	9	c,1777G>T	p.Glu593X
6	F	71	*PKD1*	Nonsense	27	c.9554G>A	p.Trp3185X
91	F	72	*PKD1*	Nonsense	20	c.7816C>T	p.Gln2606X
85	F	44	*PKD1*	Substitution	15	c.6287T>G	p.Phe2096Cys
47	M	46	*PKD1*	Substitution	37	c.10960C>G	p.Leu3654Val
12	F	50	*PKD1*	Substitution	9	c.1766T>G	p.Leu589Arg
68	F	51	*PKD1*	Substitution	23	c.8515A>T	p.Ile2839Phe
43	F	63	*PKD1*	Substitution	24	c.8819C>T	p.Pro2940Leu
8	F	73	*PKD1*	Substitution	23	c.8515A>T	p.Ile2839Phe
98	F	76	*PKD1*	Substitution	15	c.6503A>G	p.Tyr2168Cys
19	M	79	*PKD1*	Substitution	15	c.6704C>T	p.Ser2235Leu
4	M	51	*PKD2*	Large deletion	1 - 15		
122	M	44	*PKD2*	Splicing	9	c.2019+1G>T	p.Asn674fs
109	M	50	*PKD2*	Nonsense	8	c.1774C>T	p.Arg592X

13	F	61	*PKD2*	Nonsense	4	c.973C>T	p.Arg325X
86	F	73	*PKD2*	Nonsense	5	c.999T>G	p.Tyr311X

Abbreviations: RRT, renal replacement therapy; IVS, intervening sequence; M, male; F, female; ins, insertion; del, deletion
